# Physiological homeostasis, yield, and sugar profile of salt-stressed sugar beet plants as influenced by nano-structured mixture of zinc, boron, and molybdenum application

**DOI:** 10.1038/s41598-026-54565-2

**Published:** 2026-06-04

**Authors:** Eman K. Abo El-Nasr, Tawakul Y. Rizk, Hani S. Saudy, Mohamed A. H. Fergany, Mohamed A. Abd El-Hady, Ahmed Shaaban

**Affiliations:** 1 Agronomy Department, Faculty of Agriculture, Ain Shams University, Hadayek Shoubra, P.O. Box 68, Cairo, 11241 Egypt; 2 Agronomy Department, Faculty of Agriculture, Fayoum University, Fayoum, 63514 Egypt

**Keywords:** Invertase activity, Ion homeostasis, Nanotechnology, Salinization, Sugar beet physiology, Sugar quality, Physiology, Plant sciences

## Abstract

Sugar beet is a key industrial crop valued for its sucrose content, but its yield and sugar profile are greatly affected by soil salinity. In this study, we introduce novel nano-structured zinc (Zn), boron (B), and molybdenum (Mo) mixture designed to mitigate the adverse effects of salt stress. While individual nano-micronutrient applications have been investigated, the potential of combined nano-micronutrient formulations still need to be clarified. A two-season field experiments evaluated the effect of a nano-structured Zn, B, and Mo (MMNPs) mixture on salt-stressed sugar beet. Treatments included applied once (MMNPs1) or twice (MMNPs2) applications of MMNPs, their bulk counterparts (MMB1 or MMB2), and a control treatment (CK). Physiological traits, yield attributes, and sugar profile were assessed. The findings of this study pointed out that application of MMNPs2 and MMB2 treatments improved growth, photosynthetic efficiency, productivity, and juice quality compared with CK, MMNPs1, and MMB1, with MMNPs2 showing the greatest effects. Relative to CK, MMNPs2 increased net assimilation rate, absolute crop growth rate, taproot dry weight, taproot length, and taproot diameter by 3.87-, 6.14-, 1.98-, 1.24-, and 1.20-fold, respectively. It also enhanced chlorophyll *a* by 78.3%, chlorophyll *b* by 163.1%, carotenoids by 50.4%, *F*_*v*_*/F*_*₀*_ by 19.7%, *F*_*v*_*/F*_*m*_ by 3.6%, and the photosynthetic performance index by 77.7%. The MMNPs2 treatment yielded the highest sugar content and lowest non-sugar impurities. Briefly, foliar applying a nano-structured Zn, B, and Mo mixture at 165 mg L⁻¹ twice during the growth development is an effective strategy to maintain high sugar yield and quality in nutrient-deficient, sandy, salt-affected soils.

## Introduction

Sugar beet is cultivated in over 50 countries, with the European Union, particularly France and Germany, being major producers^1^, and contributes to ~ 30% of the annual 177.33 million tons of global sugar consumption^2^. In Egypt, sugar beet has overtaken sugarcane as the primary sugar source, now contributing to 61.2% (1.71 million tons) compared to sugarcane, which accounts for 29.9% of national sugar production^3^. The Egyptian newly reclaimed lands that mostly have poor sandy saline soils are the dominant regions for the sugar beet plantation^4–8^. However, depletion of micronutrients, especially zinc (Zn), boron (B), and molybdenum (Mo), is common in these soils due to intensive cropping, low organic matter content, and nutrient fixation^9–13^. This often coincides with soil salinization, a growing global problem affecting > 12% of the world’s land and > 20% of agricultural lands^14^ and ~ 25% of Egypt’s irrigated agricultural land^15^. Yearly, salinization is turning about 0.3 million hectares from once productive into degraded land, leading to considerable loss in agricultural productivity^16^.

Salinity impairs growth and yield by inducing osmotic stress, ionic toxicity due to sodium (Na^+^) and chlorine (Cl^−^) accumulation, nutrient imbalances, and oxidative damage, leading to chlorosis, reduced photosynthesis, and early senescence^17–20^. These effects together with the acute insufficiency of soil micronutrients induce cellular oxidative stress-triggered radical oxygen species (ROS) dyshomeostasis^21–24^. Cellular ROS overproduction is detrimental because it destroys biological metabolites required for cell division and differentiation^25,26^. The interference of Na^+^ and/or chloride Cl^−^ ions in protein and carbohydrate synthesis and photosynthesis leads to chlorosis and necrosis of leaves, and thus premature aging^27^. Higher Na^+^ or/and Cl^−^ concentrations not only trigger specific ion toxicity^28^, but also dyshomeostasis of the uptake of health-promoting macro- and micro-nutrients within plant cells^29^.

Micronutrients, particular zinc (Zn), boron (B), and molybdenum (Mo) play a vital role in sustaining optimum growth, development, and yield in normal and stressed conditions^10^. Many cellular metabolic pathways in plants, such as carbohydrate, lipid, and nucleic acid metabolism and biosynthesis phytohormones, such as indole-3-acetic acid and gibberellic acid need Zn element^30^. B has a crucial role in protein cytoskeletal functions, maintaining the integrity of cell wall structure and membrane^31^, phenol metabolism, hormonal regulation, cellular division and elongation, and carbohydrate transportation^32^, thereby increasing crop yield and quality^33^. Mo is a moderate or conditionally mobile micronutrient that acts as a pivotal function in the biosynthesis of chlorophyll molecules^34^ Mo can maintain configurationally and ultra-structurally the integrity of chloroplasts^35^. It is also critically involved in nitrogen (N) assimilation in sugar crops^36^, carbon and sulfur assimilatory metabolisms^37^, hormonal biosynthesis^38^, and adaptive response to environmental stresses.

However, high pH and salinity reduce micronutrients availability, making foliar application an efficient and environmentally safer alternative to soil fertilization^39–41^. High soil pH causes the essential micronutrient precipitation, including Zn^2+^ and B; however, unlike other micronutrients, the Mo in the MoO_4_^−^ form is favorable to be taken up by higher plant roots at soil pH from 5.5 up to 7.2^42,43^. Therefore, foliar fertilization compared to other field fertilization techniques (e.g., broadcast or banded) is less expensive and safer for the crop, soil, and environment^44^. The unwise broadcast or banded fertilization methods may exacerbate soil salinity problems in the long term. Thus, the multi-micronutrient fertilizers must be applied in appropriate and practicable methods, such as foliar application, particularly in nano-structured mixtures.

Nano-micronutrient mixture fertilizers are purposefully fabricated nanomaterials (1–100 nm) containing two or more micronutrients with distinct physical properties from their bulk counterparts to meet crop micronutrient needs^45^. Their unique physicochemical properties, e.g., too-tiny particle size, large specific surface area, prompt absorption, low application rates^46–48^, and less residual accumulation with easy use, make these nanomaterials excellent choics for enhancing crop yield and quality^33,49^ and reducing environmental pollution^50^.

Although numerous studies have examined the effects of single-element nano-micronutrients on crop growth, yield, and quality^51,52^, a clear knowledge gap remains regarding the efficacy of multi-element nano-structured mixtures, particularly Zn, B, and Mo, compared with their bulk counterparts under salinity and nutrient-deficient conditions. Accordingly, the objectives of this study were to compare the effects of one or two foliar application of Zn-B-Mo mixture in nano- versus bulk-form on physio-biochemical attributes, agronomic performance, yield, and sugar quality of sugar beet grown in nutrient-poor saline sandy soil.

## Materials and methods

### Study area features

Two field trials were conducted over two winter seasons: 2022/23 (October 19, 2022 – May 17, 2023) and 2023/24 (October 17, 2023 – May 15, 2024) at the Agriculture College’s experimental farm, Fayoum governorate, Egypt (29° 17’ 41.1” N, 30° 55’ 00.1” E, 32 m a.s.l.). Based on the climate classification of Köppen-Geiger^53^, the experimental site is typically characterized as a semi-arid climate with cool-wet winter and hot-dry summer. The climatic data prevailing during the 2022/23 and 2023/24 winter seasons were shown in Table 1. The routine physico-chemical soil initial characteristics at the experimental field (0–40 cm depth) following the standard procedures of Klute and Dirksen^54^ and Page et al.^55^, respectively, were illustrated in Table 2. The experimental soil was classified as sandy (75.1% sand, 13.9% silt, and 11.5% clay, averaged over the 2022/23 and 2023/24 seasons) according to the USDA^56^ classification, with inherently low water-holding capacity (WHC) and cation exchange capacity, potentially increasing nutrient leaching under irrigation.

**Table 1:** The average monthly climatic variables recorded during the 2022/23 and 2023/24 growing seasons.

Growing season	Temperature (^°^C)		RH (%)	WS (m s^− 1^)	Rainfall (mm)	Solar radiation (W m^− 2^ d^− 1^)
	Day	Night				
2022/23 season						
October	30.7	18.0	55.1	3.37	0.05	101.6
November	25.6	13.4	57.0	2.50	0.06	81.0
December	23.2	11.3	62.6	2.50	0.30	70.6
January	21.0	8.4	64.0	2.33	0.53	75.2
February	19.6	6.6	64.2	2.70	0.28	94.8
March	26.4	11.1	48.3	2.92	0.14	115.7
April	30.2	13.7	41.5	3.13	0.09	135.5
May	33.8	17.5	39.4	3.59	0.01	144.9
June	37.6	21.9	37.7	4.02	0.01	150.3
2023/24 season						
October	31.9	19.6	55.2	3.21	1.07	100.2
November	26.7	14.9	62.5	2.61	0.41	79.8
December	22.6	11.4	69.4	2.57	0.28	69.4
January	20.3	7.8	60.9	2.24	1.88	76.0
February	20.9	7.5	66.1	2.31	0.39	94.0
March	25.0	10.2	55.8	2.72	0.14	117.7
April	31.3	14.6	46.8	3.59	0.02	136.6
May	34.5	18.0	36.7	3.53	0.07	140.4
June	40.2	22.5	36.9	3.96	0.01	147.2

RH= relative humidity and WS= wind speed.

**Table 2:** Trial soil parameters (0–0.4 m depth) assessed during the 2022/23 and 2023/24 seasons.

Property	Unit	Growing season	
		2022/23	2023/24
Sand	(%)	75.0 ± 0.65	74.2 ± 0.72
Silt		13.8 ± 0.62	14.0 ± 0.46
Clay		11.2 ± 0.51	11.8 ± 0.66
Texture		Sandy loam	
Bulk density	(g cm^− 3^)	1.59 ± 0.05	1.57 ± 0.08
Hydraulic conductivity	(cm^3^ h^− 1^)	2.90 ± 0.04	2.85 ± 0.06
pH		7.79 ± 0.03	7.65 ± 0.06
EC_e_	(dS m^− 1^)	5.16 ± 0.08	4.84 ± 0.06
Cation exchange capacity	(cmole kg^− 1^)	10.81 ± 0.9	11.98 ± 1.2
CaCO_3_	(%)	5.62 ± 0.86	4.82 ± 0.66
Organic matter		0.86 ± 0.02	0.92 ± 0.01
Available nutrient			
N	(mg kg^‒1^ soil)	31.7 ± 1.9	35.1 ± 2.1
P		3.54 ± 0.7	4.08 ± 0.5
K^+^		39.2 ± 1.3	45.1 ± 1.5
Fe^2+^		15.02 ± 1.5	16.0 ± 1.8
Zn^2+^		0.50 ± 0.05	0.58 ± 0.04
B		0.32 ± 0.03	0.40 ± 0.02
Mn^2+^		20.5 ± 0.05	22.4 ± 0.04

EC_e_=electrical conductivity of the soil saturation extract.

### Experimental layout, treatments, and design

In a simple experiment, foliar micro-nutrients mixture fertilization (MMF) treatments (i.e., zinc; Zn, boron; B, and molybdenum; Mo) effects on physio-biochemical and agronomic traits, yield, and technological sugar quality traits of sugar beet grown under nutrient-poor saline sandy soil conditions were assessed. The foliar MMF treatments were applied through once or twice spraying of micro-nutrients mixture nano form (MMNPs) and their conventional (bulk) form (MMB). These treatments were arranged in a randomized complete block design with three replications.

The applied concentrations of Zn, B, and Mo in the spray solution were 300, 150, and 45 mg L^− 1^, respectively. For MMNPs, these concentrations were reduced by two-thirds to 100, 50, and 15 mg L^− 1^, respectively. Distilled H₂O was used for the non-Zn-B-Mo-treated sugar beet plants, which served as the control treatment (CK). Accordingly, the foliar MMF treatments were abbreviated to control = CK, MMB1 and MMB2 = foliar spraying once and twice, respectively, with 495 mg L^− 1^ MMB, and MMNPs1 and MMNPs2 = foliar spraying once and twice, respectively, with 165 mg L^− 1^ MMNPs. The spray solutions of Zn, B, and Mo in MMB were sourced from Zn(NO_3_)_2_·6H_2_O, H₃BO₃, and NH_4_)_6_Mo_7_O_24_·4H_2_O, with a purity of 98, 99.5, and 99%, respectively, which were secured from Sigma-Aldrich (Corp. St. Louis, MO, USA). The same micro-nutrient salts were used in the chemical preparation of the spray suspension solution of the MMNPs applied in this study. All the tested MMF treatments and CK treatment were sprayed once or twice at 9–10 a.m. on sugar beet foliage to run-off spray solution drift in day-15 intervals beginning from 90 and ending at 105 days from planting (DFP). For the two growing seasons, the sugar beet’s phenological stages were monitored based on the Biologische Bundesanstalt, Bundessortenamt und CHemische Industrie (BBCH)-codifying scale^57^. Accordingly, the sugar beet plant ages when the foliar MMF treatments synchronized with BBCH 34/35 (principal growth stage 3: leaves cover 40–50% of the ground), and BBCH 36/37 (principal growth stage 3: leaves cover 60–70% of the ground) stages. To ensure maximum permeation of MMF solution into sugar beet shoot, a few drops of 0.2% (*v/v*) tween-20 were added to each treatment solution using a 20-liter knapsack atomizer.

### Preparation and characterization of nano-micro-nutrients

**Fig. 1:**
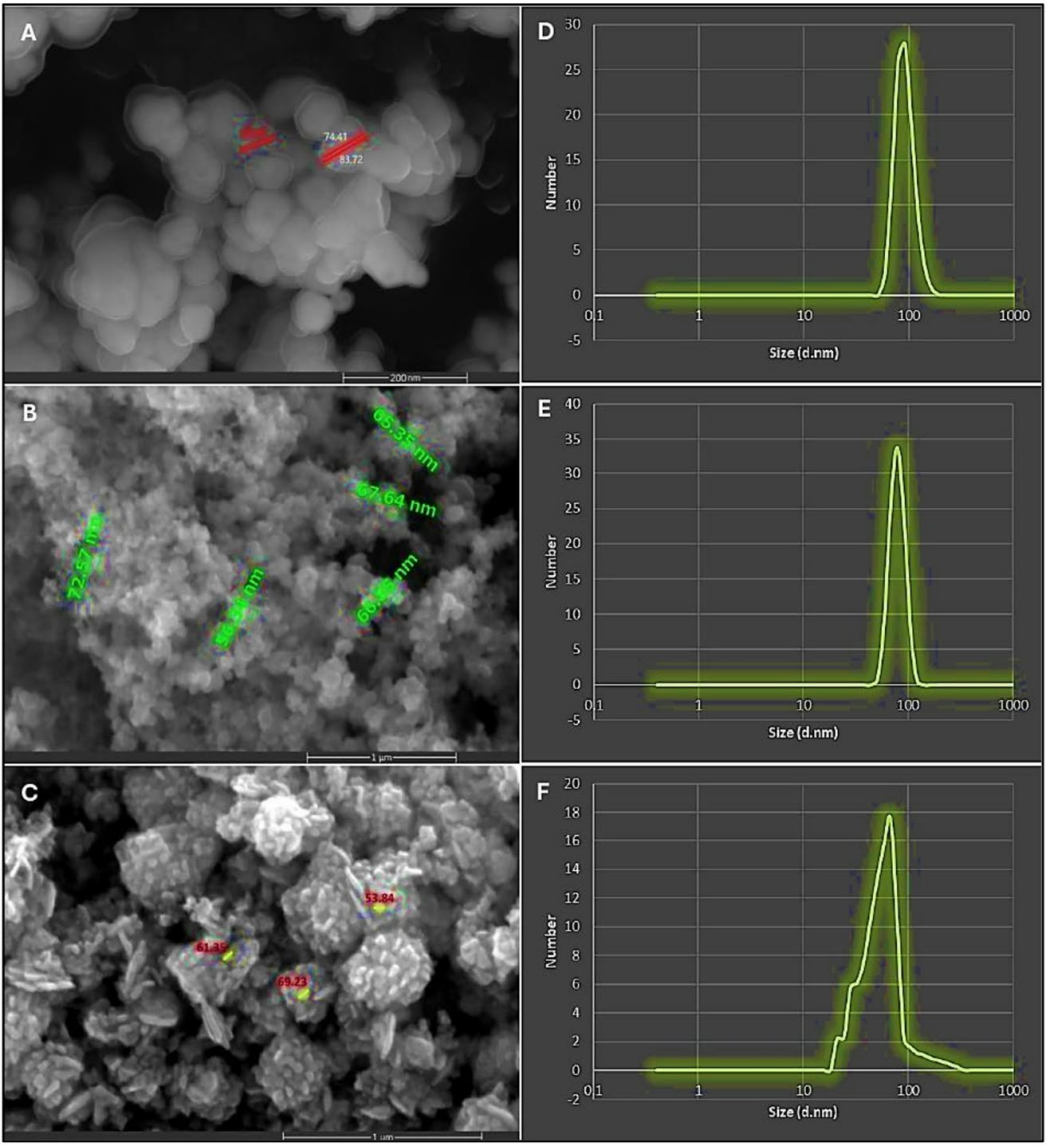
Surface morphology of the prepared (**A**) zinc (Zn), (**B**) boron (**B**), and (**C**) molybdenum (Mo) nanoparticles (NPs) and their particle size distribution graphs by dynamic light scattering (DLS) technique; where (**D**) DLS of Zn-NPs, (**E**) DLS of B-NPs, and (**F**) DLS of Mo-NPs.

Zinc oxide (ZnO) nanoparticles were synthesized using the co-precipitation protocol originally described by Sabir et al.^58^, with slight modifications by Abou El-Nasr et al.^59^. Boron oxide (B₂O₃) nanoparticles were prepared following the procedure of Allam et al.^60^ with modifications, while molybdenum trioxide (MoO₃) nanoparticles were synthesized as described by Pradeesh et al.^61^. For characterization of nano-Zn, B, or Mo micro-nutrient mixture (Fig. 1A-F), surface morphology of nanomaterials was examined by a Quattro S environmental scanning electron microscope (Thermo Fisher Scientific, Waltham, MA, USA) instrument with acceleration voltage of 5–30 kV. Well-dried samples were carefully sectioned, then fixed on specific grids. Particle size distribution was measured by dynamic light scattering (ZS nano, Malvern Pan Analytical, Westborough, MA, USA).

### Field and crop management

During the two seasons, monogerm sugar beet (*Beta vulgaris* L. cv. BTS 435) was bred by GmbH Betaseed Inc. (Frankfurt am Main, Germany) and kindly provided by the Sugar Crop Research Institute, Agricultural Research Centre (ARC), Giza, Egypt. The plant material used in this study was cultivated and not collected from the wild; hence, no special collection permits or ethical permissions were required. All experimental procedures involving plant materials were carried out following institutional, national, and international guidelines. The field experiments complied fully with the regulations and guidelines set by the ARC, Egypt.

Seeds were sown on October 19, 2022 and October 17, 2023. Each plot included 5 furrows 0.6 m wide and 4 m long, forming a 12 m^2^ net plot area. Manual sowing of seeds (3–4 seeds, having 99.9% germination) was done in hills on one side of the planting ridge with an inter-row spacing of 0.15 m. To maintain one healthy plant per hill, the sugar beet seedlings were uniformly thinned by hand at BBCH 13/14 (principal growth stage 1: 3 to 4 unfolded leaves) to achieve an approximately plant density of 111.07 thousand ha^− 1^. Sugar beet plants in all treatments were fertilized with N, P_2_O_5_, and K_2_O with rates of 288, 72, and 58 kg ha^− 1^, respectively, as locally recommended for newly reclaimed sand soil^62^. In this regard, during the experimental field preparation before sowing, the full P_2_O_5_ fertilizer amount in calcium superphosphate (12% P_2_O_5_) form and 20% of N fertilizer amount in ammonium nitrate (33.5% N) form were added as a basal fertilizer. The remaining 80% of the N fertilizer amount was divided into two equal doses, the first was manually top-dressed after the thinning process along with the full K_2_O fertilizer amount in potassium sulfate (48% K_2_O) form, and the second was applied a month later. Weeds were controlled via application of manual hoeing twice 21 and 35 days after sowing. The sugar beet water requirements, during both growing seasons, were fully supplied by the surface irrigation furrow method according to the recommendations of field irrigation management with water salinity of 0.46 dS m^− 1^.

### Assessments

#### Growth, physiological performance and nutritional status

At 120 corresponded to BBCH 39/40, 10 guarded sugar beet plants with intact taproots were taken randomly per experimental plot to measure taproot-shoot growth characteristics and physio-biochemical indices and mineral nutrients content.

#### Growth indices

Once the sugar beet plants were uprooted, they were transported instantly to the laboratory to count the leaf number plant^− 1^. According to Wallace and Munger^63^ with slight modifications, the plant leaf area (PLA in dm^2^) was measured, and then leaf area index (LAI) was calculated^64^. Sugar beet plants were separated into tops and taproots and were weighed to obtain the fresh weights of top, taproot, and whole plant. The taproot length and taproot diameter were gauged. Dry weights of top, taproot, and whole plant were measured after drying at 80 °C for about a week in a forced-air oven till constant weight. The plant shoot/taproot (Sh/TR) ratio percentage was calculated on a dry weight (DW) basis.

Further samples were taken at 150 DFP, corresponding to BBCH 43/44, to assess sugar beet growth and biomass allocation indices, involving specific leaf weight (SLW mg DW cm^− 2^), net assimilation rate (NAR mg DW cm^− 2^ d^− 1^) and absolute crop growth rate (ACGR g DW d^− 1^), of sugar beet plants between 120 and 150 DFP were calculated by growth indices of Hunt^65^.

#### Leaf photosynthetic pigments and photosynthetic performance

Using the *N*,* N*-dimethylformamide (DMF) solvent, the amounts of chlorophylls (chlorophyll *a* and chlorophyll *b*, and total carotenoid in fully expanded sugar beet leaves were measured using Lichtenthaler and Buschmann^66^ method. To assess leaf photosynthetic performance, the chlorophyll *a* fluorescence was measured with a Portable Handy PEA Chlorophyll Fluorimeter (Hansatech, Kings Lynn, UK). Accordingly, the fast fluorescence kinetics from the minimum (*F*_*0*_) to maximum (*F*_*m*_) fluorescence of the fully dark-adapted leaf area were measured. *F*_*m*_-*F*_*0*_ calculated the maximum variable chlorophyll *a* fluorescence (*F*_*v*_), while maximum PSIΙ’s photochemical efficiency [*F*_*v*_*/F*_*m*_= (*F*_*m*_-*F*_*0*_) ÷ *F*_*m*_] and PSII’s potential photochemical activity (*F*_*v*_*/F*_*0*_) were calculated as per Maxwell and Johnson^67^ from the measured parameters. The photosynthetic performance index (PI), which indicates electron flux from PSII to the reduction of intersystem electron acceptors, was measured as per Clark et al.^68^.

#### Sugars profile, total phenols (TPs), and soluble acid-invertase (SA-Inv) enzyme activity

The total sugars and reducing sugars (RS) content (g 100 g^− 1^ fresh weight; FW) in beet leaves and taproots were determined following the Shaffer-Hartman iodometric titration standard method^69^. The non-reducing sugars (NRS) content (g 100 g^− 1^ FW) was calculated by subtraction of the reducing sugars from the total sugars. The TPs content in sugar beet leaves and taproots was determined according to the standard method of Singleton and Rossi^70^. Following the method outlined in Tang et al.^71^, the SA-Inv enzyme activity (U g^− 1^ FW) was assessed.

#### Mineral nutrients in sugar beet shoot

The macro-nutrient (e.g., N, phosphorus; P, potassium; K) concentration (%) and micro-nutrient (e.g., Zn, B, and Mo) content (mg kg^− 1^ DW) were determined in sugar beet leaves at 120 DFP, respectively. The total N content was determined using the micro-Kjeldahl classical method^72^. Total P content was determined as per the protocol detailed by Watanabe and Olsen^73^. The content of K^+^, Zn^2+^, B, and Mo was determined following the method of Jones^74^ using a Spectro CIROS^CCD^ model inductively coupled plasma-mass spectrometry (Spectro Analytical Instruments, Kleve, Germany).

#### Sugar beet yield and technological sugar quality

At 210 DFP, all sugar beet plants from the two central planting ridges with an area of 4.8 m^2^, manually harvested and cleaned. Plants were then weighted to obtain the biological yield (t ha^− 1^), while taproots were separated to obtain taproot yield (TRY in t ha^− 1^). Further, harvest index (HI%) was calculated by dividing taproot yield by biological yield multiplied by 100.

A representative taproot sample from each treatment was washed, processed to mush, and flash-frosted at − 20 °C until sent to the commercial sugar technology laboratory of Sugar Industrialization Company, Egypt, where their sugar quality were measured. The sucrose concentration (SC%) was estimated polarimetrically as outlined in ICUMSA^75^ standard method using an Automatic Sugar Polarimetric Apparatus. The content (mmol 100 g^− 1^ root) of non-sucrose impurities, i.e., juice sodium (Na^+^) and juice potassium (K^+^) was determined using a flame photometric method, whereas the juice alpha-amino-N (α-A-N) was measured spectrophotometrically using a copper blue number method^76,77^. The extractable sugar concentration (ESC%) was computed as follows: ESC%= SC% - [0.343 (K^+^ + Na^+^) + (0.0939 × α-A-N) + 0.29]. Sucrose lost to molasses (SLM%) = SC% - ESC%^78^. The sugar yield in t ha^− 1^ was calculated by multiplying TRY by SC%. Extractable sugar yield (ESY; t ha^− 1^) was calculated by multiplying TRY by ESC%. Juice purity percentage was calculated according to Devillers^79^: Purity (%) = 99.36 – [14.27 (Na^+^ + K^+^ + α-A-N as meq 100 g^− 1^) ÷ SC%)].

### Statistical data analysis

The obtained data were analyzed according RCBD with 3 replicates. A combined analysis of variance (ANOVA) was performed for all studied variables in the two experimental seasons using INFOSTAT version 2020 software statistical program (Córdoba University, Córdoba, Argentina). Combining cropping seasons for analysis was based on the statistically acceptable error variance homogeneity (F-test) test for all studied variables. For post-ANOVA mean separation, the Duncan’s test comparisons were applied at *p* ≤ 0.05.

## Results

### Characterization of nano-Zn, B, and Mo-micronutrients

Micrographs obtained by scanning electron microscopy identified the morphology of nano-Zn, B, or Mo micronutrients (Fig. 1A-C), which clearly showed monodispersed uniform particle size with a spherical shape. On the other hand, particle size distribution for each Zn, B, or Mo in nano-form was measured by dynamic light scattering technique, and the results demonstrated that the average Zn, B, or Mo particle size distribution was found to be 91.3, 78.8, and 68.1 nm, respectively (Fig. 1D-F).

### Growth indices

Foliar MMF significantly (*p* ≤ 0.01) influenced the growth parameters in sugar beet plants grown under nutrient-poor saline (5.0 dS m^− 1^) sandy soil conditions compared CK (Table 3) at 120 DFP. Both bulk (MMB1 and MMB2) and nano-form (MMNPs1 and MMNPs2) foliar MMF treatments increased LAI value by 7.3–67.2% (*p* ≤ 0.01), with MMNPs2, achieving the highest LAI value. Plant DW rose progressively across treatments (from MMB1 to MMNPs2,) achieved a 31.8–90.6% enhancement over CK. The Sh/TR ratio significantly (*p* ≤ 0.01) decreased in all foliar MMF treatments compared to CK, indicating better biomass allocation to taproots. The SLW increased notably in MMB1 and MMNPs1 by 14.6 and 11.1%, respectively but showed inconsistent responses in other treatments.

**Table 3:** Leaf area index, dry weight shoot/taproot ratio, and specific leaf weight of salt-stressed sugar beet plants as influenced by zinc, boron and molybdenum mixture in nano and bulk forms (data pooled over 2022/23 and 2023/24 winter seasons).

Treatment	Leaf area index	Plant dry weight (g)	Shoot/taproot ratio	Specific leaf weight (mg cm^− 2^)
CK	4.39 ± 0.17d	82.6 ± 11.1e	0.63 ± 0.14a	6.93 ± 0.16c
MMB1	4.71 ± 0.28d	108.9 ± 4.1d	0.49 ± 0.09bc	7.94 ± 0.41a
MMB2	5.84 ± 0.28c	117.0 ± 5.5c	0.50 ± 0.07b	7.11 ± 6.90bc
MMNPs1	6.81 ± 0.22b	138.6 ± 5.4b	0.49 ± 0.05bc	7.70 ± 0.55ab
MMNPs2	7.34 ± 0.08a	157.4 ± 3.0a	0.45 ± 0.05c	7.21 ± 0.44bc

CK= Control, spraying with distilled H_2_O. MMB1 and MMB2 = foliar spraying once and twice, respectively, with zinc (300 mg L^− 1^), boron (150 mg L^− 1^), and molybdenum (45 mg L^− 1^) mixture in conventional bulk form. MMNPs1 and MMNPs2 = foliar spraying once and twice, respectively, with zinc (100 mg L^− 1^), boron (50 mg L^− 1^), and molybdenum (15 mg L^− 1^) mixture in nano form. Different letters of columns exhibit that there are significant variations at 0.05 level of probability. Means were distinguished employing Duncan’s multiple range test (*p ≤ 0.05*).

Foliar application of MMF significantly (*p* ≤ 0.01) enhanced biomass accumulation indices in sugar beet plants compared to the control (Table 4), with nano-formulated MM sprayed twice (MMNPs2) at one-third (165 mg L^− 1^) of bulk MM, exhibiting superior efficacy over MMB. MMNPs2 was the efficient treatment for improving biomass accumulation indices, surpassing the other MMF and CK treatments. The increases in NAR, ACGR, taproot DW, taproot length and taproot diameter due to MMNPs2 application amounted to 3.87, 6.14, 1.98, 1.24 and 1.20-fold, respectively, greater than CK.

**Table 4:** Net assimilation rate (NAR), Absolute crop growth rate (ACGR) and taproot traits of salt-stressed sugar beet plants as influenced by zinc, boron and molybdenum mixture in nano and bulk forms (data pooled over 2022/23 and 2023/24 winter seasons).

Treatment	NAR (mg DW cm^− 2^ d^− 1^)	ACGR (g DW d^− 1^)	Taproot	Length (cm)	Diameter (cm)
			Dry weight (g)		
CK	0.16 ± 0.02d	1.71 ± 0.24d	55.4 ± 11.6d	14.9 ± 0.20d	16.5 ± 1.30d
MMB1	0.19 ± 0.02 cd	2.30 ± 0.13 cd	75.1 ± 7.20c	15.3 ± 0.07d	17.7 ± 1.10c
MMB2	0.24 ± 0.02bc	3.28 ± 0.27bc	79.7 ± 7.30c	16.3 ± 0.11c	18.2 ± 1.20c
MMNPs1	0.29 ± 0.04b	4.48 ± 0.62b	93.7 ± 6.10b	17.2 ± 0.14b	18.8 ± 1.30b
MMNPs2	0.62 ± 0.16a	10.5 ± 2.70a	109.8 ± 5.83a	18.5 ± 0.35a	19.8 ± 1.40a

CK= Control, spraying with distilled H_2_O. MMB1 and MMB2 = foliar spraying once and twice, respectively, with zinc (300 mg L^− 1^), boron (150 mg L^− 1^), and molybdenum (45 mg L^− 1^) mixture in conventional bulk form. MMNPs1 and MMNPs2 = foliar spraying once and twice, respectively, with zinc (100 mg L^− 1^), boron (50 mg L^− 1^), and molybdenum (15 mg L^− 1^) mixture in nano form. Different letters of columns exhibit that there are significant variations at 0.05 level of probability. Means were distinguished employing Duncan’s multiple range test (*p ≤ 0.05*).

### Leaf photosynthetic pigments and photosynthetic performance

Table 5 showed that foliar MMF significantly (*p* ≤ 0.01) enhanced sugar beet’s photosynthetic parameters compared to CK. Nano-formulated MM (MMNPs1/MMNPs2, sprayed at one-third the concentration of bulk MM) distinctly outperformed MMB1 or MMB2 treatment, demonstrating formulation type (bulk vs. nano) and dosage-dependent efficacy. Relative to CK, all foliar MMF treatments increased photosynthetic pigments and photochemical efficiency indices, with the highest improvements in Chl *a* by 74.0 or 78.3%, Chl *b* by 131.0 or 163.1%, carotenoids by 38.8 or 50.4%, *F*_*v*_*/F*_*₀*_ by 16.3 or 19.7%, *F*_*v*_*/F*_*m*_ by 3.4 or 3.6%, and PI by 75.4 and 77.7% for MMNPs1- or MMNPs2-treated sugar beet plants, respectively.

**Table 5:** Plant pigments and photosynthetic efficiency of salt-stressed sugar beet plants as influenced by zinc, boron and molybdenum mixture in nano and bulk forms (data pooled over 2022/23 and 2023/24 winter seasons).

Treatment	Plant pigment (mg cm^− 2^)			Photosynthetic efficiency		
	Chlorophyll a	Chlorophyll b	Carotenoids	F_v_/F_0_	F_v_/F_m_	PI
CK	44.2 ± 1.65d	16.8 ± 1.24e	13.9 ± 1.30c	5.02 ± 0.05d	0.83 ± 0.01c	8.61 ± 0.42d
MMB1	74.2 ± 0.64c	27.3 ± 2.20d	18.9 ± 0.17b	5.52 ± 0.23c	0.85 ± 0.01b	11.5 ± 1.33c
MMB2	76.4 ± 1.03b	33.8 ± 0.43c	19.5 ± 0.24b	5.75 ± 0.16b	0.85 ± 0.01b	12.7 ± 0.77bc
MMNPs1	76.9 ± 1.78b	38.8 ± 0.41b	19.3 ± 0.15b	5.84 ± 0.13b	0.85 ± 0.01b	15.1 ± 0.57b
MMNPs2	78.8 ± 2.04a	44.2 ± 0.45a	20.9 ± 0.70a	6.01 ± 0.04a	0.86 ± 0.01a	15.3 ± 0.48a

CKCK= Control, spraying with distilled H_2_O. MMB1 and MMB2 = foliar spraying once and twice, respectively, with zinc (300 mg L^− 1^), boron (150 mg L^− 1^), and molybdenum (45 mg L^− 1^) mixture in conventional bulk form. MMNPs1 and MMNPs2 = foliar spraying once and twice, respectively, with zinc (100 mg L^− 1^), boron (50 mg L^− 1^), and molybdenum (15 mg L^− 1^) mixture in nano form. *F*_*v*_*/F*_*0*_= photosystem II’s potential photochemical activity, *F*_*v*_*/F*_*m*_= maximum photosystem II’s photochemical efficiency, *F*_*v*_ = variable fluorescence, *F*_*m*_ = maximum fluorescence, *F*_*0*_= initial fluorescence, and PI= performance index. Different letters of columns exhibit that there are significant variations at 0.05 level of probability. Means were distinguished employing Duncan’s multiple range test (*p ≤ 0.05*).

### Sugars profile, total phenols (TPs), and soluble acid-invertase (SA-Inv) activity

Foliar MMF significantly (*p* ≤ 0.05 or *p* ≤ 0.01) affected sugars profile, TPs, and SA-Inv activity in sugar beet plants grown under nutrient-poor saline (5.0 dS m^− 1^) sandy soil conditions (Table 6). The MMB and MMNPs treatments exhibited a dose-dependent trend (MMB2 > MMB1 and MMNPs2 > MMNPs1), with intermediate and superior efficacy over the unsprayed control treatment, respectively. In shoot, MMNPs_1_ or MMNPs_2_ significantly (*p* ≤ 0.01) increased total sugars by 32.3% or 40.5%, NRS by 43.9% or 53.7%, NRS/RS ratio by 8.4 and 9.6%, and TPs by 38.3% or 40.3%, respectively, while reducing SA-Inv activity by 50.2% or 58.7% (*p* ≤ 0.01). Similarly, in taproot organ, MMNPs1 and MMNPs2 significantly (*p* ≤ 0.01) elevated total sugars by 37.1 and 45.9%, NRS by 47.1 and 55.7%, NRS/RS ratio by 6.8 and 6.8%, and TPs by 37.2 and 215% (*p* ≤ 0.05). Notably, the total sugars, NRS, and NRS/RS ratio in both the shoot and taproot organs of sugar beet, in addition to the TPs in the shoot, at MMNPs2 were statistically comparable to those obtained at MMNPs1 treatment.

**Table 6:** Total sugars, non-reducing sugars (NRS), non-reducing sugars/reducing sugars (NRS/RS) ratio and total phenols (TPs) in shoot and taproot of salt-stressed sugar beet plants as influenced by zinc, boron and molybdenum mixture in nano and bulk forms (data pooled over 2022/23 and 2023/24 winter seasons).

Treatment	Total sugars (g 100 g^− 1^)	NRS (g 100 g^− 1^)	NRS/RS ratio (g 100 g^− 1^)	TPs (mg g^− 1^)
		Shoot		
CK	8.54 ± 0.26c	7.09 ± 0.26c	0.83 ± 0.01c	31.3 ± 13.1c
MMB1	9.30 ± 0.38bc	7.92 ± 0.33bc	0.85 ± 0.01bc	33.0 ± 13.3bc
MMB2	9.81 ± 0.36b	8.46 ± 0.37b	0.86 ± 0.01b	36.4 ± 13.4b
MMNPs1	11.3 ± 0.44a	10.2 ± 0.44a	0.90 ± 0.01a	43.3 ± 15.3a
MMNPs2	12.0 ± 0.34a	10.9 ± 0.28a	0.91 ± 0.01a	43.9 ± 15.0a
		Taproot		
CK	7.95 ± 0.16c	7.00 ± 0.09c	0.88 ± 0.01c	19.1 ± 7.97b
MMB1	8.51 ± 0.34c	7.71 ± 0.34c	0.91 ± 0.01b	22.7 ± 9.90b
MMB2	9.34 ± 0.35b	8.62 ± 0.39b	0.92 ± 0.01ab	24.5 ± 10.5b
MMNPs1	10.9 ± 0.30a	10.3 ± 0.28a	0.94 ± 0.01a	26.2 ± 15.0b
MMNPs2	11.6 ± 0.25a	10.9 ± 0.24a	0.94 ± 0.01a	60.2 ± 27.6a

CK= Control, spraying with distilled H_2_O. MMB1 and MMB2 = foliar spraying once and twice, respectively, with zinc (300 mg L^− 1^), boron (150 mg L^− 1^), and molybdenum (45 mg L^− 1^) mixture in conventional bulk form. MMNPs1 and MMNPs2 = foliar spraying once and twice, respectively, with zinc (100 mg L^− 1^), boron (50 mg L^− 1^), and molybdenum (15 mg L^− 1^) mixture in nano form. Different letters of columns exhibit that there are significant variations at 0.05 level of probability. Means were distinguished employing Duncan’s multiple range test (*p ≤ 0.05*).

### Mineral nutrients in sugar beet shoot

Foliar application of MMF significantly (*p* ≤ 0.01) raised nutrient content in sugar beet’s shoot at 120 DFP under nutrient-poor saline (5.0 dS m^− 1^) conditions compared to CK, with MMNPs1 and MMNPs2 treatments, demonstrating superior efficacy over the MMB1 or MMB2 (Table 7). Both macro-(N, P, and K) and micro (Zn, B, and Mo)-nutrients exhibited noticeable increases with MMNPs2, achieving the greatest content of N (133.6% increase over CK), P (25.0% increase over CK), K^+^ (15.2% increase over CK), Zn^2+^ (208.2% increase over CK), B (185.3% increase over CK), and Mo (56.7% increase over CK). While MMB1/MMB2 treatments improved mineral nutrient content, their effects at triple the concentration (495 mg L^− 1^) were consistently lower than nano treatments at one-third (165 mg L^− 1^) concentration, underscoring the enhanced nutrient uptake efficiency of nano-formulated micro-nutrients.

**Table 7:** Leaf macro- and micro-nutrients of salt-stressed sugar beet plants as influenced by zinc, boron and molybdenum mixture in nano and bulk forms (data pooled over 2022/23 and 2023/24 winter seasons).

Treatment	Macro-nutrient (%)			Micro-nutrient (mg kg^− 1^)		
	Nitrogen	Phosphorus	Potassium	Zinc	Boron	Molybdenum
CK	1.13 ± 0.03c	0.16 ± 0.01b	1.12 ± 0.01b	21.9 ± 4.1c	20.4 ± 0.1c	14.1 ± 0.1c
MMB1	2.06 ± 0.06b	0.19 ± 0.01a	1.13 ± 0.01b	35.3 ± 2.6b	34.2 ± 2.5b	16.0 ± 0.5c
MMB2	2.48 ± 0.06a	0.19 ± 0.01a	1.26 ± 0.02a	55.0 ± 4.0a	54.0 ± 3.9a	19.8 ± 2.2b
MMNPs1	2.63 ± 0.09a	0.20 ± 0.01a	1.27 ± 0.01a	61.8 ± 4.4a	56.1 ± 4.0a	20.9 ± 2.2ab
MMNPs2	2.64 ± 0.07a	0.20 ± 0.01a	1.29 ± 0.01a	67.5 ± 4.4a	58.2 ± 4.1a	22.1 ± 2.2a

CK= Control, spraying with distilled H_2_O. MMB1 and MMB2 = foliar spraying once and twice, respectively, with zinc (300 mg L^− 1^), boron (150 mg L^− 1^), and molybdenum (45 mg L^− 1^) mixture in conventional bulk form. MMNPs1 and MMNPs2 = foliar spraying once and twice, respectively, with zinc (100 mg L^− 1^), boron (50 mg L^− 1^), and molybdenum (15 mg L^− 1^) mixture in nano form. Different letters of columns exhibit that there are significant variations at 0.05 level of probability. Means were distinguished employing Duncan’s multiple range test (*p ≤ 0.05*).

### Sugar beet yield and technological sugar quality

**Fig. 2:**
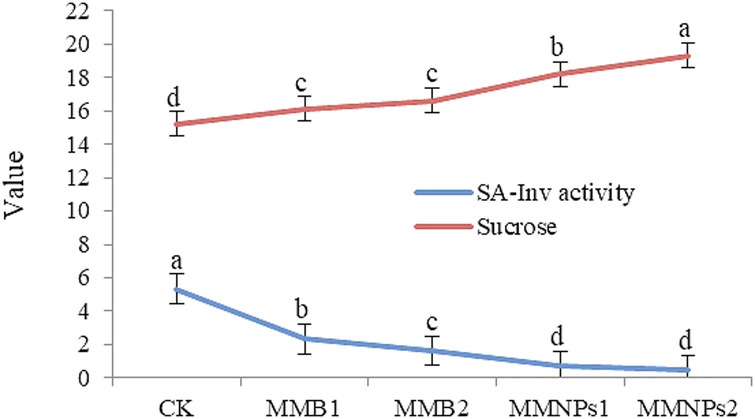
Soluble acid-invertase (SA-Inv) activity (U g^− 1^ FW) and sucrose concentration (%) of salt-stressed sugar beet plants as influenced by zinc, boron and molybdenum mixture in nano and bulk forms (single vs. double spray). CK= Control, spraying with distilled H_2_O. MMB1 and MMB2 = foliar spraying once and twice, respectively, with zinc (300 mg L^− 1^), boron (150 mg L^− 1^), and molybdenum (45 mg L^− 1^) mixture in conventional bulk form. MMNPs1 and MMNPs2 = foliar spraying once and twice, respectively, with zinc (100 mg L^− 1^), boron (50 mg L^− 1^), and molybdenum (15 mg L^− 1^) mixture in nano form. Data pooled over 2022/23 and 2023/24 winter seasons. Different letters exhibit that there are significant variations at 0.05 level of probability. Means were distinguished employing Duncan’s multiple range test (*p ≤ 0.05*).

Compared to CK, all foliar MMF treatments significantly dose-dependently enhanced sugar beet yield performance, with the highest enhancements observed under MMNPs2 (Table 8). The TRY rose progressively from 104.8 up to 143.4 t ha^− 1^ for MMB_1_ to MMNPs2 treatments, respectively, reflecting a 48.4% gain over CK. Sugar yield followed a similar trend, rising by 89.0%, while ESY rose increased by100.9% with MMNPs2. The HI on a taproot fresh matter basis improved from 78.5% in CK to 90.9% in the MMNPs2-treated crop plants, achieving a 15.8% improvement. Contrastingly, the SA-Inv activity (Fig. 2) decreased gradually, particularly with repeated spraying of nano-formulations, dropping from 5.34 U g^− 1^ FW in CK to 0.51 U g^− 1^ FW in MMNPs2. This diminution was inversely correlated with SC (Fig. 2), which steadily rose and peaked at 19.3% under MMNPs2 compared to 15.2% in CK, pointing to lower enzymatic sucrose inversion in the sugar beet taproot.

**Table 8:** Yields and harvest index (HI) of salt-stressed sugar beet plants as influenced by zinc, boron and molybdenum mixture in nano and bulk forms (data pooled over 2022/23 and 2023/24 winter seasons).

Treatment	Yield (t ha^− 1^)			HI (%)
	Taproot	Sugar	Extractable sugar	
CK	96.6 ± 0.70e	14.6 ± 0.35e	10.9 ± 0.34e	78.5 ± 0.28e
MMB1	104.8 ± 1.1d	16.9 ± 0.57d	12.8 ± 0.56d	83.3 ± 0.18d
MMB2	113.8 ± 1.6c	18.9 ± 0.54c	14.6 ± 0.50c	85.2 ± 0.38c
MMNPs1	129.6 ± 1.1b	23.6 ± 0.44b	18.3 ± 0.52b	88.3 ± 0.39b
MMNPs2	143.4 ± 0.88a	27.6 ± 0.42a	21.9 ± 0.31a	90.9 ± 0.23a

CK= Control, spraying with distilled H_2_O. MMB1 and MMB2 = foliar spraying once and twice, respectively, with zinc (300 mg L^− 1^), boron (150 mg L^− 1^), and molybdenum (45 mg L^− 1^) mixture in conventional bulk form. MMNPs1 and MMNPs2 = foliar spraying once and twice, respectively, with zinc (100 mg L^− 1^), boron (50 mg L^− 1^), and molybdenum (15 mg L^− 1^) mixture in nano form. Different letters of columns exhibit that there are significant variations at 0.05 level of probability. Means were distinguished employing Duncan’s multiple range test (*p ≤ 0.05*).

**Fig. 3:**
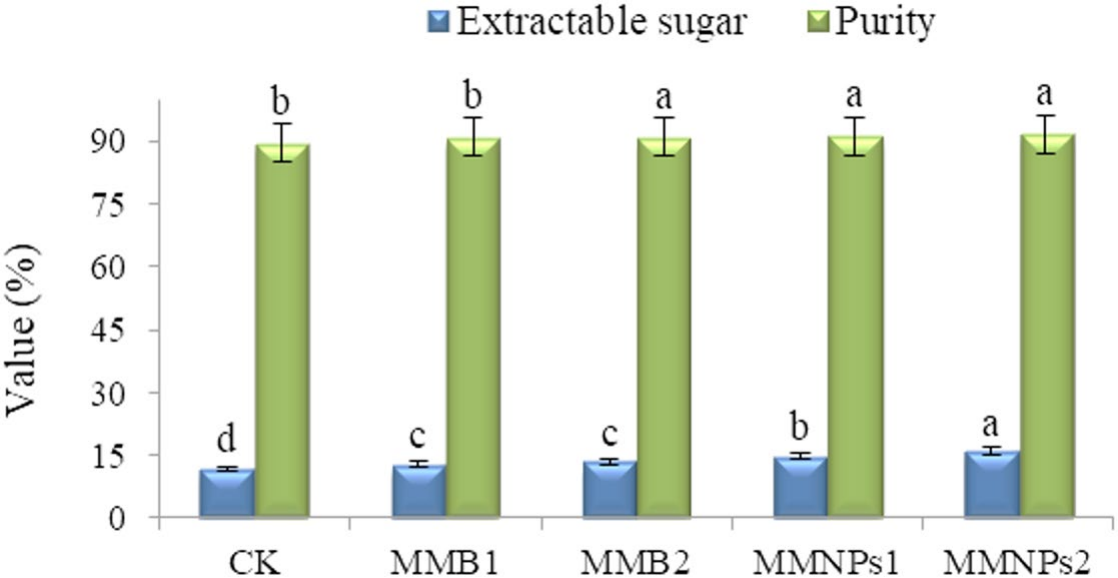
Extractable sugar concentration (%) and purity (%) of salt-stressed sugar beet plants as influenced by zinc, boron and molybdenum mixture in nano and bulk forms (single vs. double spray). CK = CK= Control, spraying with distilled H_2_O. MMB1 and MMB2 = foliar spraying once and twice, respectively, with zinc (300 mg L^− 1^), boron (150 mg L^− 1^), and molybdenum (45 mg L^− 1^) mixture in conventional bulk form. MMNPs1 and MMNPs2 = foliar spraying once and twice, respectively, with zinc (100 mg L^− 1^), boron (50 mg L^− 1^), and molybdenum (15 mg L^− 1^) mixture in nano form. Data pooled over 2022/23 and 2023/24 winter seasons. Different letters of bars exhibit that there are significant variations at 0.05 level of probability. Means were distinguished employing Duncan’s multiple range test (*p ≤ 0.05*).

Foliar MMF treatments differentially influenced technological sugar quality (Table 9), with nano-formulated MM at one-third the concentration (MMNPs1 or MMNPs2) of bulk forms, demonstrating their superior efficacy, despite the juice K⁺, juice α-A-N, and SLM remaining unaffected. Compared to the control-treated plants (CK), foliar MMNPs2 significantly increased ESC by 35.6% and sugar purity by 2.3% (Fig. 3). While, MMB2 reduced juice Na⁺ content by 12.0%. Foliar application of MMB1 or MMB2 treatment enhanced ESC, but only MMB2 improved sugar purity significantly.

**Table 9:** Non-sucrose impurities and sucrose lost to molasses (SLM) in juice of salt-stressed sugar beet plants as influenced by zinc, boron and molybdenum mixture in nano and bulk forms (data pooled over 2022/23 and 2023/24 winter seasons).

Treatment	Non-sucrose impurities (mmol 100 g^− 1^ root)			SLM (%)
	Na^+^	K^+^	α-A-*N*	
CK	3.85 ± 0.46ab	4.46 ± 0.48a	2.07 ± 0.10a	3.33 ± 0.08a
MMB1	4.14 ± 0.45a	4.27 ± 0.36a	2.02 ± 0.12a	3.36 ± 0.10a
MMB2	3.39 ± 0.79b	4.39 ± 0.36a	1.87 ± 0.28a	3.13 ± 0.20a
MMNPs1	4.09 ± 0.44a	4.20 ± 0.31a	2.06 ± 0.21a	3.33 ± 0.09a
MMNPs2	4.22 ± 0.37a	3.75 ± 0.19a	2.39 ± 0.08a	3.25 ± 0.08a

CK= Control, spraying with distilled H_2_O. MMB1 and MMB2 = foliar spraying once and twice, respectively, with zinc (300 mg L^− 1^), boron (150 mg L^− 1^), and molybdenum (45 mg L^− 1^) mixture in conventional bulk form. MMNPs1 and MMNPs2 = foliar spraying once and twice, respectively, with zinc (100 mg L^− 1^), boron (50 mg L^− 1^), and molybdenum (15 mg L^− 1^) mixture in nano form. Na^+^= sodium, K^+^= potassium, and α-A-N = α-amino nitrogen. Different letters of columns exhibit that there are significant variations at 0.05 level of probability. Means were distinguished employing Duncan’s multiple range test (*p ≤ 0.05*).

## Discussion

Salinity combined with nutrient deficiency severely limits plant physio-biochemical and agronomic performance, ultimately reducing crop yield and quality-related attributes^80–84^. Given the sandy texture of the experimental soil, nutrient retention and WHC were inherently low, which could intensify leaching losses under irrigation. This characteristic likely amplified the importance of nano-formulated micronutrients, whose tiny particle size and high surface reactivity can improve nutrient uptake and reduce losses, thereby mitigating the constraints of saline, sandy, nutrient-deficient soils.

As expected, the present study revealed that nutrient-poor saline conditions depressed sugar beet physiology, biomass allocation, and yield (Tables 3, 4, 5, 6, 7 and 8). These findings therefore support the need for targeted fertilization strategies to correct micronutrient deficiencies in saline sandy soils^85–87^. Exogenous foliar application of a Zn-B-Mo mixture alleviated salinity-induced impairments, with the nano-formulation applied twice (MMNPs2) producing the greatest overall improvements in physiological performance, nutrient status, sugar accumulation, and yield (Tables 3, 4, 5, 6, 7, 8 and 9). These benefits are likely achieved through improved foliar uptake and internal mobilization of micronutrients under saline conditions^88^.

Mechanistically, Zn, B, and Mo micronutrients operate complementarily to sustain membrane integrity and photosynthetic performance and promote antioxidant defense, hormone synthesis, and carbohydrate metabolism under salt stress^89–91^. Our findings corroborate that Zn initiates CO_2_ fixation in the Calvin-Benson cycle and co-catalysts key metalloenzymes, e.g., fructose 1,6-biphosphatase, carbonic anhydrase (CAase), and ribulose 1,5-diphosphate carboxylase/oxygenase, which drive this process^92,93^. Further, since Zn had a significant role in stimulating indole-3-acetic acid synthesis^39^, improved plant growth was noticed with Zn supply. It has been reported that Zn dramatically contributes to protecting the thylakoid structure from ROS injuries via stimulation of superoxide dismutase production, thus arming the plant against salinity^94^. Furthermore, the enhancement of photosynthetic pigments and PSII efficiency might be interpreted by the involvement of Zn in stimulating CAase activity^95^.

Salinity reduced pigment content and growth indices, while the micronutrient mixture partially restored these parameters by maintaining leaf relative water content and membrane stability^96–98^. Foliar application with a Zn-B-Mo mixture stimulated the phyto-production of cytokinins, gibberellins, auxins, phenols, and amino acids in sugar beet leaves, facilitating the accumulation of photosynthetic products, mainly sugars, in the treated plants^99–101^. Elevating phenol levels refer to strengthening the tolerance to stresses that could substantially decline the growth and yield^102^. This outcome may be attributed to increased polyphenol oxidase activity^103^, likely driven by healthy growth and enhanced sugar sink capacity from efficient photosynthesis and active photoassimilate transport to sugar beet roots.

Boron supplementation likely supported pigment biosynthesis and PSII efficiency in agreement with Abd El-Hady^104^, who found that increasing B caused a significant increase in leaf chlorophyll *a* and *b* content and carotenoids of sugar beet. These results could be attributed to the fact that B is an essential element for photosynthetic pigments, thus enhancing photosynthetic O_2_ photoevolution and CO_2_ fixation and consequently upgrading PSII electron transport efficiency^105^. Moreover, B may have maintained thylakoid membrane integrity by decreasing Cl⁻ uptake and increasing Cl⁻ exudation^106^, thereby improving the photosynthetic process in sugar beet^32^.

Similarly, Mo application improved pigment levels, consistent with its role in chlorophyll biosynthesis and chloroplast structural stability, in agreement with Zewail et al.^100^, who reported that foliar applying Mo to sugar beet plants led to chlorophyll *a* and *b* and carotenoids. This result indicates that Mo might have a vital role in the chlorophyll biosynthesis pathway and chloroplast integrity in terms of configuration and ultrastructure^107^. According to Yu et al.^108^, the conversion of δ-aminolaevulinic acid to uroporphyrinogen III, an intermediate in the chlorophyll biosynthesis route, takes place in the chloroplast structure rather than on thylakoid membranes during the chlorophyll biosynthesis route. This process is halted in the absence of Mo, which lowers the amount of chlorophyll in the leaf tissues. The robust chloroplast configuration and ultrastructure are positively associated with chlorophyll biosynthesis and the PSII light-harvesting complex^109^.

This study confirmed salinity-induced reductions in sugar beet yields (taproot, sugar, and extractable sugar), HI, and technological sugar quality, while nano- or bulk-form micronutrient treatments significantly improved these traits (Tables 8 and 9; Figs. 2 and 3). As for sugar beet juice quality, the current research divulged that the Zn-B-Mo mixture in bulk or nano form augmented SC, ESC, and purity while reducing in SA-Inv enzyme activity. However, the micronutrient applications did not show clear changes in juice Na^+^, K^+^, α-A-N, and SLM. As a result of enhancing K absorption through the application of micronutrients, the quality characteristics of sugar beet juice were expected to be improved. It has been reported that K acts as a key role in the transport of photosynthetic substances (sucrose) from source organs to sink organs^110^. Since it is involved in the activation of carbohydrate metabolism enzymes, Zn supplementation has shown a potential effect in increasing sucrose^93^. Despite higher leaf K in treated plants, juice K decreased, likely due to its retention in leaves as part of salinity tolerance mechanisms^111^.

The superior performance of the nano-form mixture over the bulk form reflects the nanoscale particle’s greater solubility, larger surface area due to their smaller size, and effective cellular uptake^112^. This mechanistic advantage likely underlies MMNPs2’s greater stimulation of photosynthesis and antioxidant activation, and ion homeostasis (Tables 5, 6, 7 and 8), as reflected in our physiological and yield attributes relative to bulk forms^113^. For instance, it can quicken the rate of photosynthesis by enhancing the formation of plant pigments compared to bulk particles^114^. Accordingly, the current research demonstrated that the nano-micronutrient mixture was more efficient than the conventional nutrient form in stimulating sugar beet physiology, growth, yield, and quality.

The synergistic benefit of the nano-formulated multi-element mixture likely arises from the coordinated uptake and interactive roles of Zn, B, and Mo at the cellular level^89,90^. In nanoscale mixtures, the close physical association of different micronutrient particles can facilitate co-delivery and concurrent absorption through foliar cuticles and stomatal apertures, thereby reducing the competitive antagonism often observed when elements are supplied separately^115^. Following internalization, Zn facilitates enzymatic activation and antioxidant fortification, B reinforces cell wall cross-linking and carbohydrate translocation, while Mo is indispensable for nitrate reduction and chlorophyll biosynthesis, collectively promoting integrated metabolic restoration under salinity stress^116^. This “multi-nano” approach has been reported to improve nutrient-use efficiency, optimize photosynthetic capacity, and strengthen stress resilience more effectively than single-element nanoparticles^91^. Our findings corroborate and extend these observations to sugar beet grown in saline soil, demonstrating that the functional complementarity of Zn, B, and Mo mixture in nano-form optimizes physiological homeostasis and ensures yield stability.

## Conclusions

The present investigation establishes that foliar application of a nano-structured zinc, boron, and molybdenum mixture at 165 mg L^− 1^, applied twice during crop development, is an effective strategy to enhance sugar beet performance under salinity stress. The novelty of this approach lies in its integrated modulation of photosynthetic efficiency, nutrient homeostasis, and post-harvest sugar metabolism, resulting in improved sugar content, greater purity, and fewer non-sucrose impurities. These findings highlight that multi-micro-element nano-fertilizers can simultaneously optimize the agronomic and yield potentiality, and industrial sugar quality under nutrient-deficient sandy soil conditions in salinity-prone areas.

## Data Availability

The datasets used and/or analyzed during the present investigation available from the corresponding author on reasonable request.

## Abbreviations

Zn: Zinc
B: Boron
Mo: Molybdenum
CK: Control treatment
MMNPs: Micronutrients mixture in nano-particles form
MMB: Micronutrients mixture in bulk form
NAR: Net assimilation rate
ACGR: Absolute crop growth rate
MMF: Micro-nutrients mixture fertilization
Sh/TR: Shoot/taproot ratio
DFP: Days from planting
DMF: N, N-dimethylformamide
BBCH: Biologische Bundesanstalt, Bundessortenamt und CHemische Industrie
PLA: Plant leaf area
LAI: Leaf area index
*F*_*v*_/*F*_*m*_: Photochemical efficiency
*F*_*v*_/*F*_*0*_: PSII’s potential photochemical activity
PI: Photosynthetic performance index
SA-Inv: Soluble acid-invertase activity
TPss: Total phenols
HI: Harvest index
NRS: Non-reducing sugars
SC: Sucrose concentration
SLM: Sucrose lost to molasses
TRY: Taproot yield
ESC: Extractable sugar concentration
ESY: Extractable sugar yield
DLS: Dynamic light scattering
